# Enhancement of cellulosome-mediated deconstruction of cellulose by improving enzyme thermostability

**DOI:** 10.1186/s13068-016-0577-z

**Published:** 2016-08-04

**Authors:** Sarah Moraïs, Johanna Stern, Amaranta Kahn, Anastasia P. Galanopoulou, Shahar Yoav, Melina Shamshoum, Matthew A. Smith, Dimitris G. Hatzinikolaou, Frances H. Arnold, Edward A. Bayer

**Affiliations:** 1 Department of Biomolecular Sciences, The Weizmann Institute of Science, 76100 Rehovot, Israel; 2 Microbiology Group, Faculty of Biology, National and Kapodistrian University of Athens, Zografou Campus, 15784 Athens, Greece; 3 Faculty of Agricultural, Food and Environmental Quality Sciences, The Hebrew University of Jerusalem, P.O. Box 12, 76100 Rehovot, Israel; 4 Division of Chemistry and Chemical Engineering, California Institute of Technology, Pasadena, CA 91125 USA; 5 Department of Biological Chemistry, The Weizmann Institute of Science, 76100 Rehovot, Israel

**Keywords:** Thermostable cellulases, Multi-enzyme complex, Designer cellulosomes, *Clostridium thermocellum*

## Abstract

**Background:**

The concerted action of three complementary cellulases from *Clostridium thermocellum,* engineered to be stable at elevated temperatures, was examined on a cellulosic substrate and compared to that of the wild-type enzymes. Exoglucanase Cel48S and endoglucanase Cel8A, both key elements of the natural cellulosome from this bacterium, were engineered previously for increased thermostability, either by SCHEMA, a structure-guided, site-directed protein recombination method, or by consensus-guided mutagenesis combined with random mutagenesis using error-prone PCR, respectively. A thermostable β-glucosidase BglA mutant was also selected from a library generated by error-prone PCR that will assist the two cellulases in their methodic deconstruction of crystalline cellulose. The effects of a thermostable scaffoldin versus those of a largely mesophilic scaffoldin were also examined. By improving the stability of the enzyme subunits and the structural component, we aimed to improve cellulosome-mediated deconstruction of cellulosic substrates.

**Results:**

The results demonstrate that the combination of thermostable enzymes as free enzymes and a thermostable scaffoldin was more active on the cellulosic substrate than the wild-type enzymes. Significantly, “thermostable” designer cellulosomes exhibited a 1.7-fold enhancement in cellulose degradation compared to the action of conventional designer cellulosomes that contain the respective wild-type enzymes. For designer cellulosome formats, the use of the thermostabilized scaffoldin proved critical for enhanced enzymatic performance under conditions of high temperatures.

**Conclusions:**

Simple improvement in the activity of a given enzyme does not guarantee its suitability for use in an enzyme cocktail or as a designer cellulosome component. The true merit of improvement resides in its ultimate contribution to synergistic action, which can only be determined experimentally. The relevance of the mutated thermostable enzymes employed in this study as components in multienzyme systems has thus been confirmed using designer cellulosome technology. Enzyme integration via a thermostable scaffoldin is critical to the ultimate stability of the complex at higher temperatures. Engineering of thermostable cellulases and additional lignocellulosic enzymes may prove a determinant parameter for development of state-of-the-art designer cellulosomes for their employment in the conversion of cellulosic biomass to soluble sugars.

**Graphical abstract:**
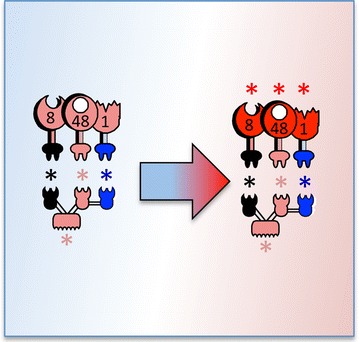
Conversion of conventional designer cellulosomes into thermophilic designer cellulosomes

**Electronic supplementary material:**

The online version of this article (doi:10.1186/s13068-016-0577-z) contains supplementary material, which is available to authorized users.

## Background

One of the most efficient approaches for degradation of plant cell-wall polysaccharides (notably cellulose) in Nature is the integration of cellulases and associated enzymes into an extracellular, generally cell-associated, multi-enzymatic complex named the cellulosome [1]. Cellulosome architecture consists of a non-catalytic “scaffoldin” subunit and two complementary recognition modules termed “dockerin” and “cohesin” that together serve to integrate the various enzymes into the complex [2, 3]. In *Clostridium thermocellum*, the most extensively studied cellulosome producer, the non-catalytic primary scaffoldin subunit comprises a string of 9 repeating cohesin modules, plus a single carbohydrate-binding module (CBM) and an X-dockerin modular dyad that interacts with an anchoring scaffoldin at the cell surface [4]. Cellulosomal enzymes contain a catalytic module and a divergent type of dockerin module, which binds tightly to the cohesins of the primary scaffoldin, thereby effecting their integration into the complex.

We have employed the designer cellulosome strategy as a conceptual platform for promoting synergistic action among enzyme components [5–8]. This strategy involves the use of recombinant chimaeric scaffoldins composed of cohesin modules originating from different bacterial species, whereby each cohesin binds specifically to the matching dockerin of the same species, harbored by the different enzymes. These artificial nanodevices allow precision control of the composition and architecture of the cellulosome assembly and have proven to be efficient in their cellulolytic capacity [9–12].

In the present work, we address the question as to whether we can improve cellulosome-mediated deconstruction of cellulosic substrates by improving enzyme thermostability. Indeed, thermostable cellulolytic enzymes are particularly attractive candidates for biomass deconstruction [13]. Their resistance and robustness to high temperature can allow faster and more effective reactions (enhancement of specific activity, higher diffusion rates, higher substrate solubility, reduction in enzyme loading) [14, 15], as well as increased resistance to harsh chemical pre-treatment conditions [16–18]. Additional advantages include lower contamination risks, increased process flexibility [19, 20] and lower costs (since enzymatic reactions are exothermic, it is therefore cheaper to perform the hydrolysis at elevated temperatures, without the need for refrigeration to maintain constant temperature in the reactor) [21, 22]. Lignocellulose degradation should, thus, be carried out at temperatures above 50–55 °C [16, 23].

In previous reports we have engineered an extremely thermostable endoglucanase from *C. thermocellum* (QM, termed herein 8A*) [24, 25]. Nevertheless, the ultrastable endoglucanase displayed no advantage over the wild-type enzyme when combined with a native exoglucanase (Cel48S) in designer cellulosomes prepared for this purpose. It seems that in this case the cohesin stabilized the wild-type dockerin-containing enzyme, but failed to confer additional stability onto the inherently stabilized mutant endoglucanase [26].

In this report, we examined whether the thermostable 8A* endoglucanase would act in synergy with an enhanced thermostable exoglucanase Cel48S from *C. thermocellum* into more complex designer cellulosomes. Arnold and colleagues [27] examined 60 Cel48S mutants (each with an average of 106 mutations) obtained by shuffling 3 known GH48 catalytic modules using the SCHEMA program (a structure-guided, site-directed protein recombination method). This program identifies crossover sites for recombination of homologous proteins that maximize the likelihood that proteins in the resulting library will retain their folded structure. We selected five of these mutants and tested them before incorporation of the best performing Cel48 mutant into thermostable designer cellulosomes.

The Cel8A endoglucanase and the Cel48S exoglucanase are the most highly expressed cellulases secreted extracellularly as components of the *C. thermocellum* cellulosome complex [28]. Their action is complementary and results in high levels of synergy on the cellulosic substrate [29]. Nevertheless, their product of degradation, cellobiose, is known to induce feedback inhibition on the cellulase components [30, 31], and the integration of β-glucosidase in cellulosomes was demonstrated to enhance cellulosic substrate degradation [32]. Therefore, to ensure optimal enzymatic degradation we included a thermostable β-glucosidase generated by error-prone PCR into our thermostable designer cellulosomes. A recent study indicated that the chimaeric scaffoldin used in designer cellulosomes should also be adapted to resist at temperatures over 60 °C [33]. Therefore, we designed a novel chimaeric scaffoldin composed entirely of cohesin modules originating from thermophilic microbes. The action on microcrystalline cellulose of the resultant thermostable trivalent designer cellulosome was examined and compared to complementary conventional designer cellulosomes.

## Methods

### Cloning

Plasmid designs of BglA, 8A*, Cel48S, Cel48S*, Scaf·BTFA and Scaf·T were described previously [9, 24, 27, 32, 34, 35]. Primers for the design of BglA-*v*, BglA-v*, BglA*-*f* (in pET21a, Novagen Inc., Madison, WI), 8A-*g*, 8A*-*g*, 8A*-*b* (in pET28a, Novagen) and Scaf·GTV (in pET9d) are listed in Table 1.

**Table 1:** Primers used in the study (restriction sites represented in upper case)

Construct name (intermediate construct)	Vector	Forward primer (5’–3’)	Reverse Primer (5’–3’)	Linearized vector	Primary PCR template^a^	Secondary PCR template^a^
BglA-*v* (BglA)	pET21a	CTTTAAGAAGGAGATATACATATGGCTAGCcaccatcaccatcaccacaagataactttcccaaaaga	ccatgtaggaacgagctttgtgccACTAGTCTGAGGAGTTGTTACAGTTGTgaaaccgttgtttttgattacttc		pET28a BglA [32]	pET21a
BglA-*v*	pET21a	ttacatACTAGTtctcataaatttatctatgg	ttactaGAGCTCctattgttcttcaactggga	pET9d Xyn-docB [48]		–
BglA*-*v* (BglA*)	pET21a	CTTTAAGAAGGAGATATACATATGGCTAGCcaccatcaccatcaccacaagataactttcccaaaaga	ccatgtaggaacgagctttgtgccACTAGTCTGAGGAGTTGTTACAGTTGTgaaaccgttgtttttgattacttc		pET28a BglA*	pET21a
BglA*-*f*	pET21a	CTTTAAGAAGGAGATATACATATGGCTAGCcaccatcaccatcaccacaagataactttcccaaaaga	ccatgtaggaacgagctttgtgccACTAGTCTGAGGAGTTGTTACAGTTGTgaaaccgttgtttttgattacttc		pET28a BglA*	pET21a Xyn10A-*f* [42]
BglA*-*v*	pET21a	ttacatACTAGTtctcataaatttatctatgg	ttactaGAGCTCctattgttcttcaactggga	pET9d Xyn-docB [48]		–
8A-*g* (8A)	pET28a	ttacgtCCATGGgtgtgccttttaacacaaaata	ttacgaGGTACCaatgaaggtgtcggattcga	pET28a Cel8A [29]		–
8A-*g*	pET28a	tactagGGTACCagaagaagcaaacaagggag	ttactaCTCGAGcttacccagtaagccattct	pET28a-*g*-5A [6]		–
8A*-*g* (8A*)	pET28a	ttacgtCCATGGgtgtgccttttaacacaaaata	ttacgaGGTACCaatgaaggtgtcggattcga	pET28a 8AQM [24]		–
8A*-*g*	pET28a	tactagGGTACCagaagaagcaaacaagggag	ttactaCTCGAGcttacccagtaagccattct	pET28a-*g*-5A [6]		–
8A*-*b*	pET28a	ttacgtCCATGGgtgtgccttttaacacaaaata	ttacgaGGTACCaatgaaggtgtcggattcga	pET28a 8A-*b* [29]		
Scaf·GT	pET9d	GTTTAACTTTAAGAAGGAGATATAccatggtgcttcctccgaaaactacc	ggtgtgtttgtcggtgtgtttgtcGGTACCgcttcttcctgagagacaatc		pET28a CBM-Coh2375 [34]	pET28a Scaf·AT [49]
Scaf·GTV	pET9d	gttctttgacggtggagtaaatgttggagatacaacagtacctacaacacctacaacacctGCAGGGCAATTACAAATTGA	CCTTTCGGGCTTTGTTAGCAGCCggatcctAATTTGAGCCAACCAATATAG		pET28a CBM-CohC1 [48]	pET9d Scaf·GT

^a^Indicates restriction-free cloning

PCRs were performed with Phusion High Fidelity DNA polymerase F530-S (New England Biolabs, Inc), and PCR products and plasmids were restricted with Fastdigest enzymes (Thermo scientific, USA). T4 DNA ligase was used for ligation (Fermentas UAB, Vilnius, Lithuania). PCR products were purified using a HiYield™ Gel/PCR Fragments Extraction Kit (Real Biotech Corporation, RBC, Taiwan), and plasmids were extracted using Qiagen miniprep kit (Valencia, CA). Competent *Escherichia coli* XL1 cells were used for plasmid transformation.

### Recombinant protein expression and purification

*E. coli* BL21 (DE3) cells producing 8A-*g*, Cel48S, BglA, 8A*-*g*, 8A*-*b*, Cel48S*, BglA-v*, BglA*-*f*, Scaf·T, Scaf·GTV and Scaf·BTFA were grown in 2 L LB (Luria Broth) and 2 mM CaCl_2_ with the appropriate antibiotic at 37 °C until A_600_≈0.8–1 and induced by adding 0.1 mM (final concentration) isopropyl-1-thio-β-D-galactoside (IPTG) (Fermentas UAB Vilnius, Lithuania), and cell growth was continued at 16 °C overnight with the exceptions of 8A-*g*, 8A*-*b* and 8A*-*g* (grown 3 h at 37 °C). Cells were harvested by centrifugation at 5000 rpm for 5 min. Pelleted cells were resuspended in 30 mL TBS containing 5 mM imidazole (Tris-buffered saline, 137 mM NaCl, 2.7 mM KCl, 25 mM Tris–HCl, pH 7.4). The His-tagged enzymes were purified on a Ni–NTA column (Qiagen) as reported earlier [36], and Scaf·T, Scaf·GTV and Scaf·BTFA were purified with macroporous bead cellulose pre-swollen gel (IONTOSORB, Usti nad Labem, Czech Republic) as described previously [37]. Acrylamide gels SDS-PAGE (10 %) served to assess the purity of the recombinant proteins and absorbance at 280 nm indicated their concentrations. Extinction coefficients were estimated by the Protparam tool [38]. Proteins were stored in 50 % (v/v) glycerol at −20 °C.

### Non-denaturing gel electrophoresis

Each enzymatic component was first calibrated for its optimal ratio for full complex formation with the chimaeric scaffoldin. The three enzymes were then mixed at their optimized ratio with the scaffoldin to ensure full complex formation. Protein mixtures were supplemented with 12 mM CaCl_2_ and 0.05 % Tween 20 and incubated for 2 h at 37 °C. The electrophoretic mobility of the proteins was then analyzed by PAGE under non-denaturing conditions with gels comprising a 4.3 % stacking gel and a 9 % separation gel. Migration was carried out at 100 V. The gels were stained using InstantBlue Coomassie-based staining (Expedeon, USA).

### Enzymatic activity assay

Cellulolytic activity was tested with mixtures of free enzymes (0.5 μM each) or optimized designer cellulosomes at 0.5 μM and pH 5 (buffer acetate 50 mM final concentration) with 1 % Avicel (FMC, Delaware USA) at various incubation times and temperatures. For the kinetic experiment at 60 °C, enzymatic concentration was increased to 2.5 μM. In the case of Cel48S*, either free or in combination with Scaf·T, the concentrations of enzyme and complex were adjusted to 0.8 μM, and the reactions were incubated for 48 h at 60 °C. Enzymatic reactions were terminated by placing the tubes into an ice-water bath, and the tubes were then centrifuged for 2 min at 14,000 rpm at room temperature. Enzymatic activity was determined quantitatively by measuring soluble reducing sugars released from the cellulosic substrate by the dinitrosalicylic acid (DNS) method [39]. A volume of 150 μL of the DNS solution was added to 100 μL of sample (supernatant fluids), and after boiling the reaction mixture for 10 min, absorbance at 540 nm was measured. Released sugar concentrations were determined using a glucose standard curve. Glucose concentration was determined using a glucose assay kit (product code GAGO20; Sigma-Aldrich, Israel) according to the manufacturer’s instructions. All assays were performed at least twice in triplicate.

## Results

**Fig. 1:**
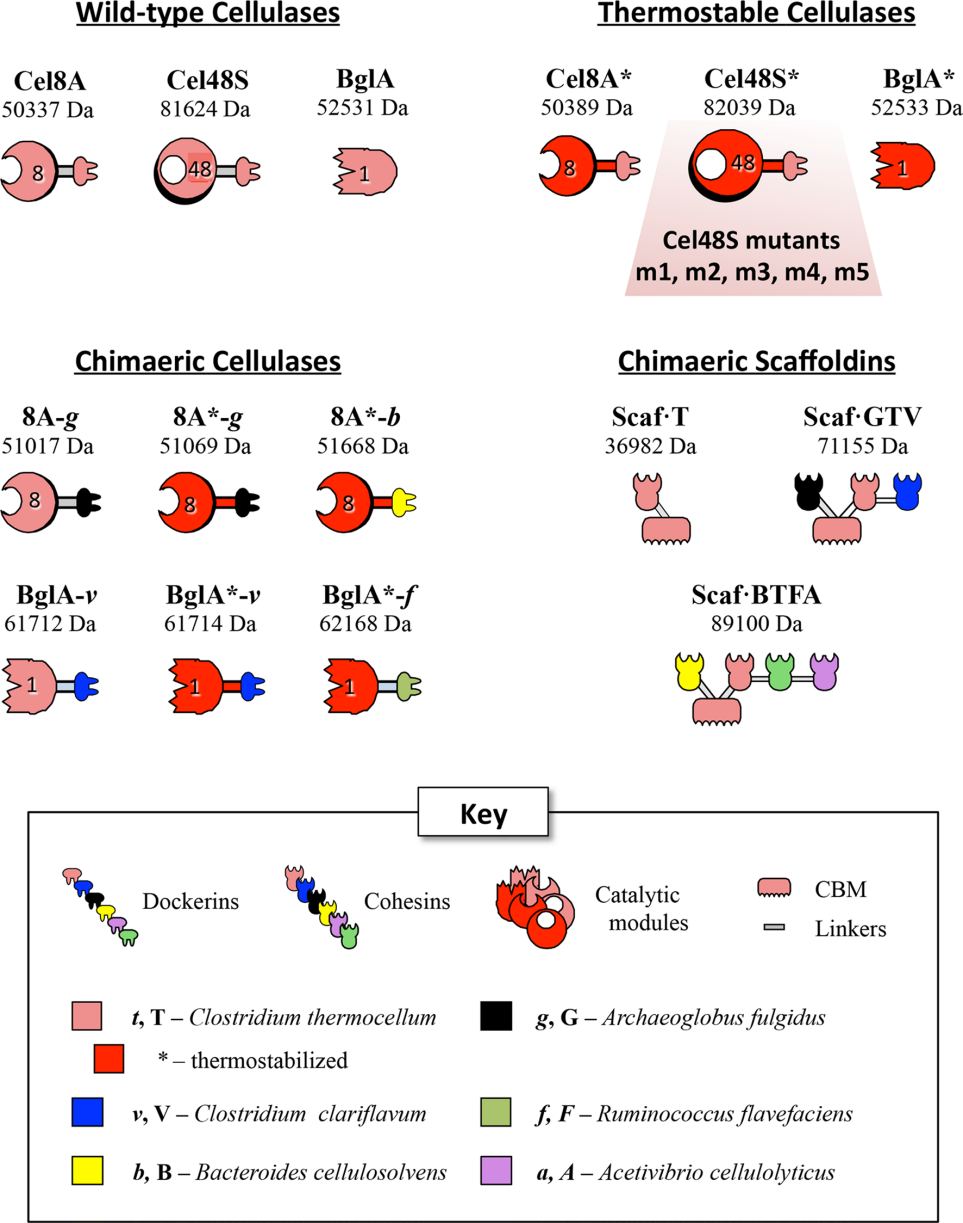
Schematic representations of the proteins used in this study. Thermostabilized forms of *C. thermocellum* catalytic modules are shown in *red pictograms* and denoted by an *asterisk*. The origin of the bacterial species from which the cohesins were acquired is shown color-coded in the key. For the purposes of this study, modular components from the thermophilic microbes, *C. thermocellum, C. clariflavum and A. fulgidus,* are considered inherently thermostable, relative to those from the mesophilic *B. cellulosolvens* and *A. cellulolyticus*

The recombinant proteins designed for use in this study are shown schematically in Fig. 1.

### Selection of thermostable Cel48S mutants

Five potent mutants of Cel48S from *C. thermocellum* that were reported to be more thermostable than the wild-type enzyme were selected from the list of 60 mutants described in a previous study by Arnold and colleagues [27]. These 5 mutants were renamed as follows: m1 = 31313333; m2 = 11312122; m3 = 23232332; m4 = 22222332; m5 = 22122332. Two of these mutants, namely m4 and m5, were originally reported to have higher specific enzymatic activity than the wild-type enzyme. Since all these mutants contained the native *C. thermocellum* dockerin, we employed the monovalent scaffoldin Scaf·T which is composed of two modules from *C. thermocellum*, i.e., the family 3a CBM, and cohesin 3 from the CipA scaffoldin (Fig. 1). This simple scaffoldin binds a single copy of either the wild-type or mutant forms of Cel48S and thus confers to them the quality of strong substrate binding. The catalytic module alone is known to bind relatively weakly to cellulose [40].

To assess precise equimolar ratios between the various designer cellulosome components, a differential mobility assay on non-denaturing gels was used (see Additional file 1). This step, which was not performed for the Cel48S mutants in the previous study [27], is crucial in order to ensure precise measurement of designer cellulosome enzymatic activity [41]. Indeed, we observed that the exact ratios between the different forms of Cel48S (wild-type and mutants) did not always correspond to the values assessed only by protein concentration measurement (an estimated 1:1 ratio is not always optimal, as can be observed in Additional file 1).

**Fig. 2:**
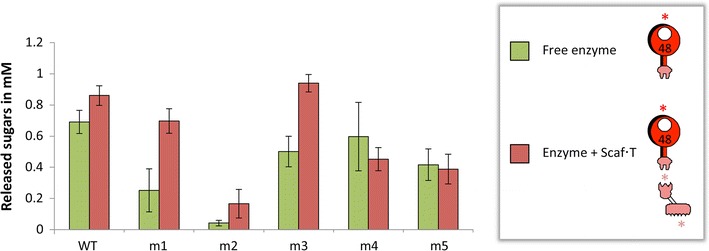
Comparative degradation of Avicel by wild-type or mutant Cel48S, either in the free state or complexed to the monovalent scaffoldin Scaf·T. Enzymatic activity is defined as mM total reducing sugars following the 48-h reaction period at 60 °C. Each reaction was performed in triplicate, and standard deviations are indicated. The *red asterisk* indicates the enhanced thermostabilized mutated form of the *C. thermocellum* Cel48S catalytic module and the *pink asterisks* indicate the inherently thermostable *C. thermocellum* cohesin and CBM

Following corrections in protein concentration of the various Cel48S derivatives, degradation of microcrystalline cellulose (or Avicel) was examined after 24- and 48-h periods of incubation at 60 °C, either in the free state (the enzyme alone) or bound to a monovalent scaffoldin Scaf·T (Fig. 2). In the free state, the wild-type enzyme demonstrated higher enzymatic activities than any of the five mutants. Nevertheless, as a component of monovalent designer cellulosomes, substrate degradation by Cel48S mutants m1 and m3 were comparable to that of the wild-type, with a slight advantage for mutant m3. Differences in enzymatic performance can be noted, however, compared to the previous study [27], presumably due to protein concentration corrections subsequent to non-denaturing PAGE mobility assay as performed in the present study. In this context, even if exoglucanase activity alone on crystalline cellulosic substrates is very low, minor variations in concentration could prove significant to synergistic action with other cellulases. We ultimately selected mutant m3 (named herein Cel48S*) for incorporation into thermostable designer cellulosomes, owing to its higher activity on Avicel than the wild-type enzyme and its increased expression in *E. coli*.

### Production of designer cellulosome components

To integrate the enzymes into designer cellulosomes in a controlled and precise manner, we had to switch the dockerin specificity of either Cel48S or Cel8A (both sharing the same *C. thermocellum* dockerin specificity). For this purpose, the original dockerin of endoglucanase Cel8A from *C. thermocellum* was replaced by the dockerin from *Archaeoglobus fulgidus* (*g*) to form 8A-*g*. The same dockerin was also appended to the thermostable mutant QM of this enzyme (termed herein 8A*) [24] to form 8A*-*g*.

In the case of β-glucosidase BglA from *C. thermocellum,* the native protein is not cellulosomal and does not include a dockerin. Therefore,to incorporate the BglA into the designer cellulosomes, we selected the ScaB dockerin from *Clostridium clariflavum* (*v*) for grafting to the C-terminus of the catalytic module to form BglA-*v*. Since there was no available wild-type linker either at the C-terminus of the *C. thermocellum* β-glucosidase or at the N-terminus of the dockerin from *C. clariflavum* ScaB, a synthetic 9 amino-acid linker (TTVTTPQTS), that bears similarity to the wild-type linker located between cohesins 4 and 5 of ScaB, was included between the two modules. The same linker and dockerin was also appended to a thermostable mutant, BglA*, generated by error-prone PCR (see Additional file 2), to form BglA*-*v*.

For the needs of this study, a novel scaffoldin, composed entirely of thermophilic modules and consistent with the complementary dockerins of the respective enzymes, was designed. Scaf·GTV is composed of three cohesin modules from three thermophilic species, *Archaeoglobus fulgidus* (G), *C. thermocellum* (T) and *Clostridium clariflavum* (V). The scaffoldin also contains a CBM3a from *C. thermocellum* for susbtrate targeting. Scaf·GTV allows the selective integration of the 3 cellulases produced in this study, i.e., Cel48S, 8A-*g* and BglA-*v,* as well as their thermostable versions, Cel48S*, 8A*-*g* and BglA*-*v*.

In order to assess the importance of the thermophilic scaffoldin Scaf·GTV, scaffoldin Scaf·BTFA of mainly mesophilic origin was used as a control for thermostability assays of the designer cellulosomes. This tetravalent scaffoldin has been described and used in earlier works [9, 33, 42] and includes four different cohesin types together with the cellulose-binding module as shown schematically in Fig. 1. From the N to C-terminus, the cohesin modules of the chimaeric scaffoldin include B from *B. cellulosolvens* for inclusion of 8A*-*b*, T from *C. thermocellum* for inclusion of Cel48S*, F from *Ruminococcus flavefaciens* for inclusion of BglA*-*f* and A from *Acidothermus cellulolyticus* which will remain unoccupied, with a potent cellulose-binding *C. thermocellum* family-3a carbohydrate-binding module (CBM3a) positioned between B and T.

### Thermostability of the designer cellulosome complexes

**Fig. 3:**
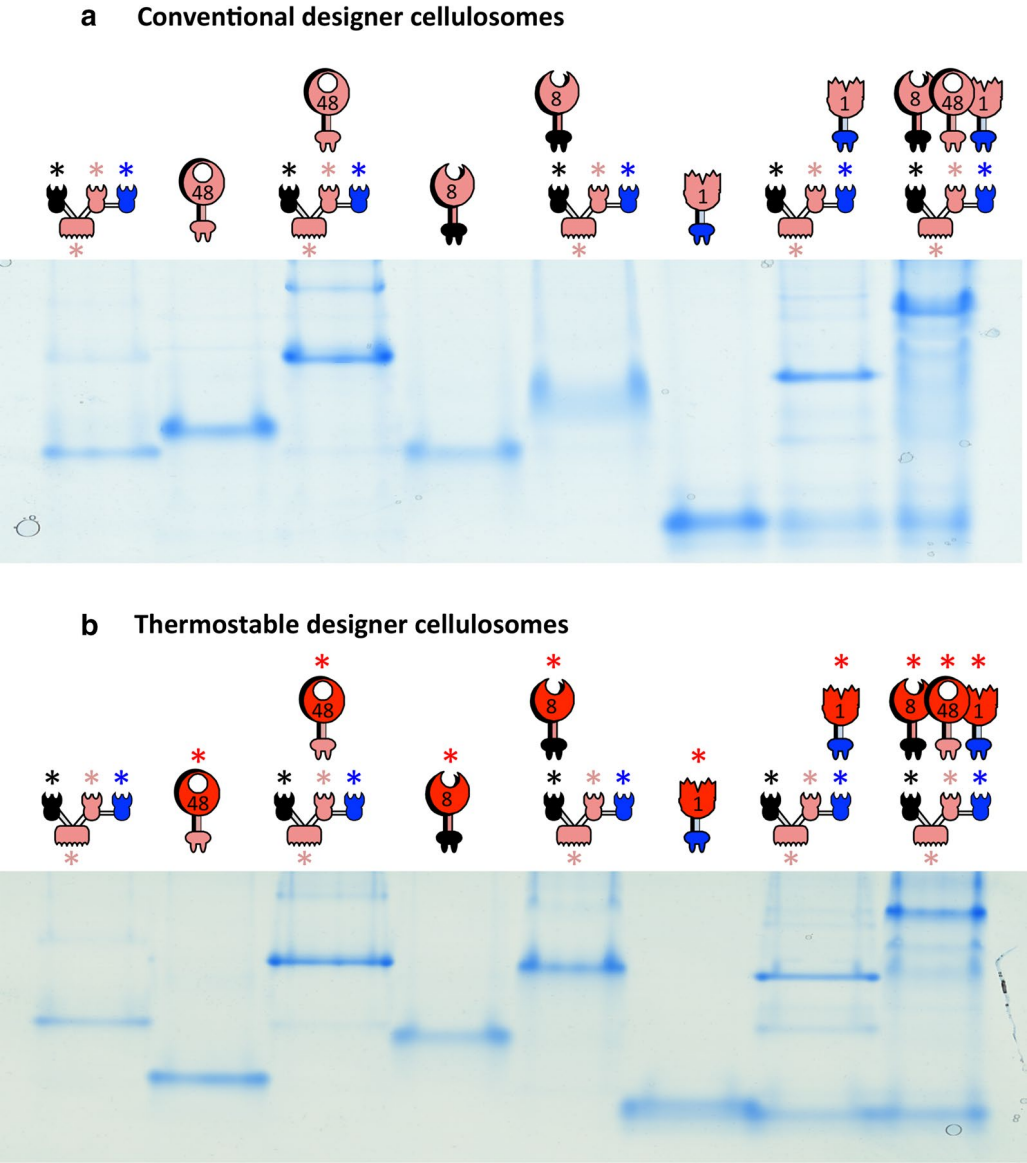
Electrophoretic mobility of components and assembled complexes as assessed by non-denaturing gels. Equimolar concentrations of the chimeric enzymes and their matching scaffoldin were combined to form **a** conventional designer cellulosomes or **b** thermostable designer cellulosomes. Near-complete interaction is indicated by the formation of a shifted major band. Wild-type forms of the *C. thermocellum* modules (Cel48S, Cel8A and Bgl1A catalytic modules, cohesin and CBM of the chimaeric scaffoldin) are shown in *pink pictograms*. Thermostabilized mutated forms of the respective catalytic module are shown in* red pictograms* and denoted by an *asterisk*. The *pink*, *black* and *blue asterisks* indicate the inherently thermostable *C. thermocellum* cohesin and CBM, *A. fulgidus* and *C. clariflavum* cohesins, respectively

**Fig. 4:**
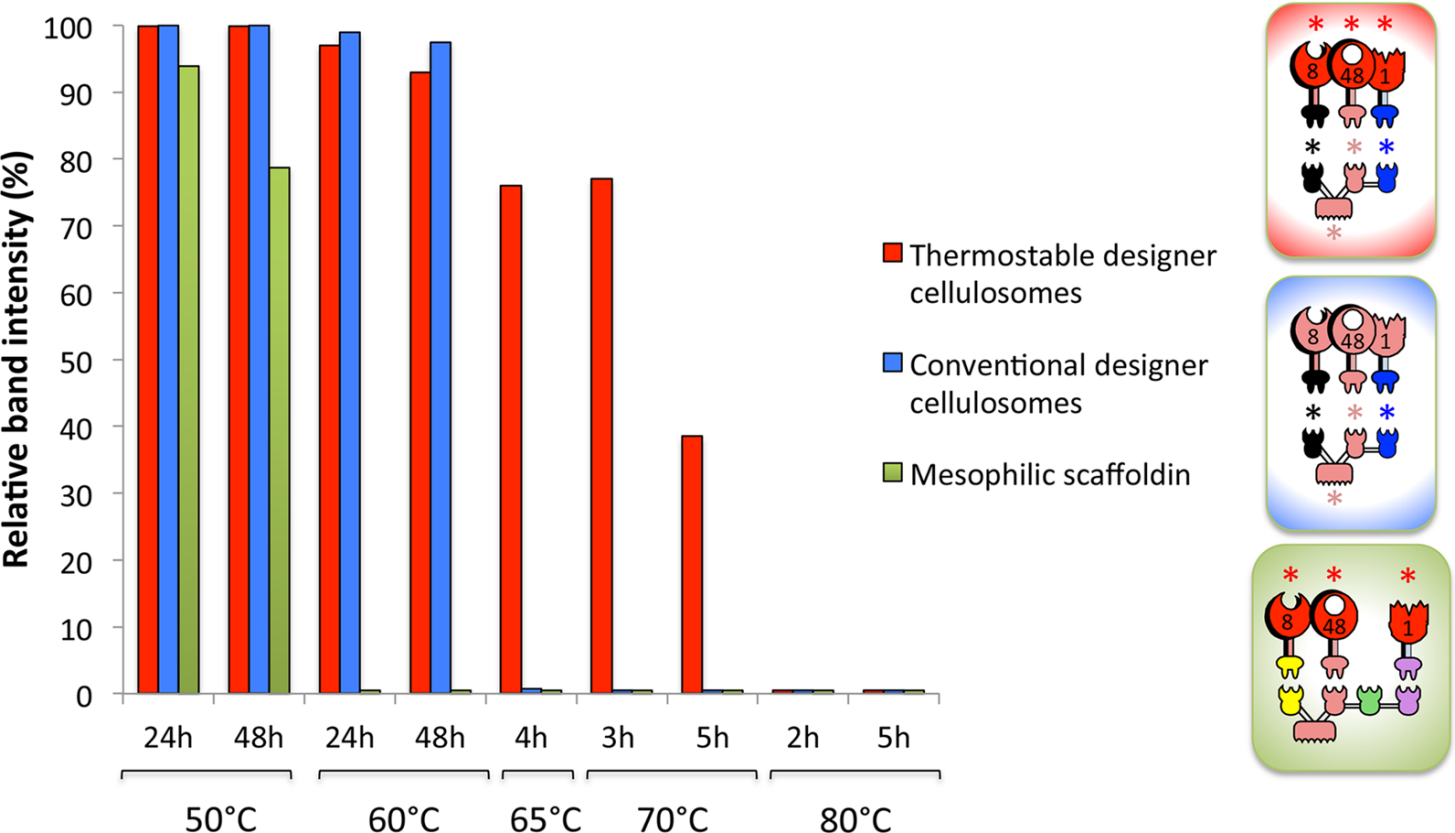
Densitometric analysis for the determination of thermal stabilities of thermostable designer cellulosomes (*red bars*), conventional designer cellulosomes (*blue bars*) and designer cellulosomes with the mesophilic scaffoldin (*green bars*) at different times and incubation temperatures. Band image densities were determined by Adobe Photoshop CS5. Pictograms denote composition of the three designer cellulosomes. See legend for Fig. 3 for definitions and descriptions of color schemes and *asterisks*

The ratio of each component of the designer cellulosomes was optimized by non-denaturing PAGE, as described above for Cel48S and the chimaeric monovalent scaffoldin Scaf·T (data not shown). We thus assembled designer cellulosome complexes, containing either the wild-type enzymes or the thermostable mutants (Fig. 3) and compared their stability at elevated temperatures. Both types of complex proved to be stable at 50 and 60 °C for 48 h. The thermostable designer cellulosomes were also stable for 4 h at 65 °C and 3 h at 70 °C, as opposed to the conventional designer cellulosomes comprising the corresponding wild-type enzymes (see Fig. 4 and Additional file 3A, B).

As a control, the three thermostable (Additional file 3C) enzymes in complex with Scaf·BTFA of mainly mesophilic origin were incubated at various temperatures and different incubation times. The complex proved stable only for 48 h at 50 °C (Fig. 4 and Additional file 3D), which is in accordance with a recent report in which a designer cellulosome containing Scaf·BTFA was stable for only 6 h at 60 °C [33].

The stability of the trivalent scaffoldin either alone or in complex with each of the thermostable dockerin-containing enzymes was also assayed (Additional file 3D). The results revealed that the scaffoldin alone was stable for 40 h at 70 °C, but when complexed to the dockerin-containing enzymes, the scaffoldin underwent denaturation when incubated more than 3 h at this temperature. Only 8A*-*g* attached to the scaffoldin was more stable than the other single enzymes attached to the scaffoldin (i.e., Cel48S* and BglA*-*v*). The complex was still present in the non-denaturing gel after 4 h of incubation at 70 °C, suggesting that the 8A*-g is the most thermostable enzyme of the three enzymes tested.

### Enzymatic activities of designer cellulosomes

**Fig. 5:**
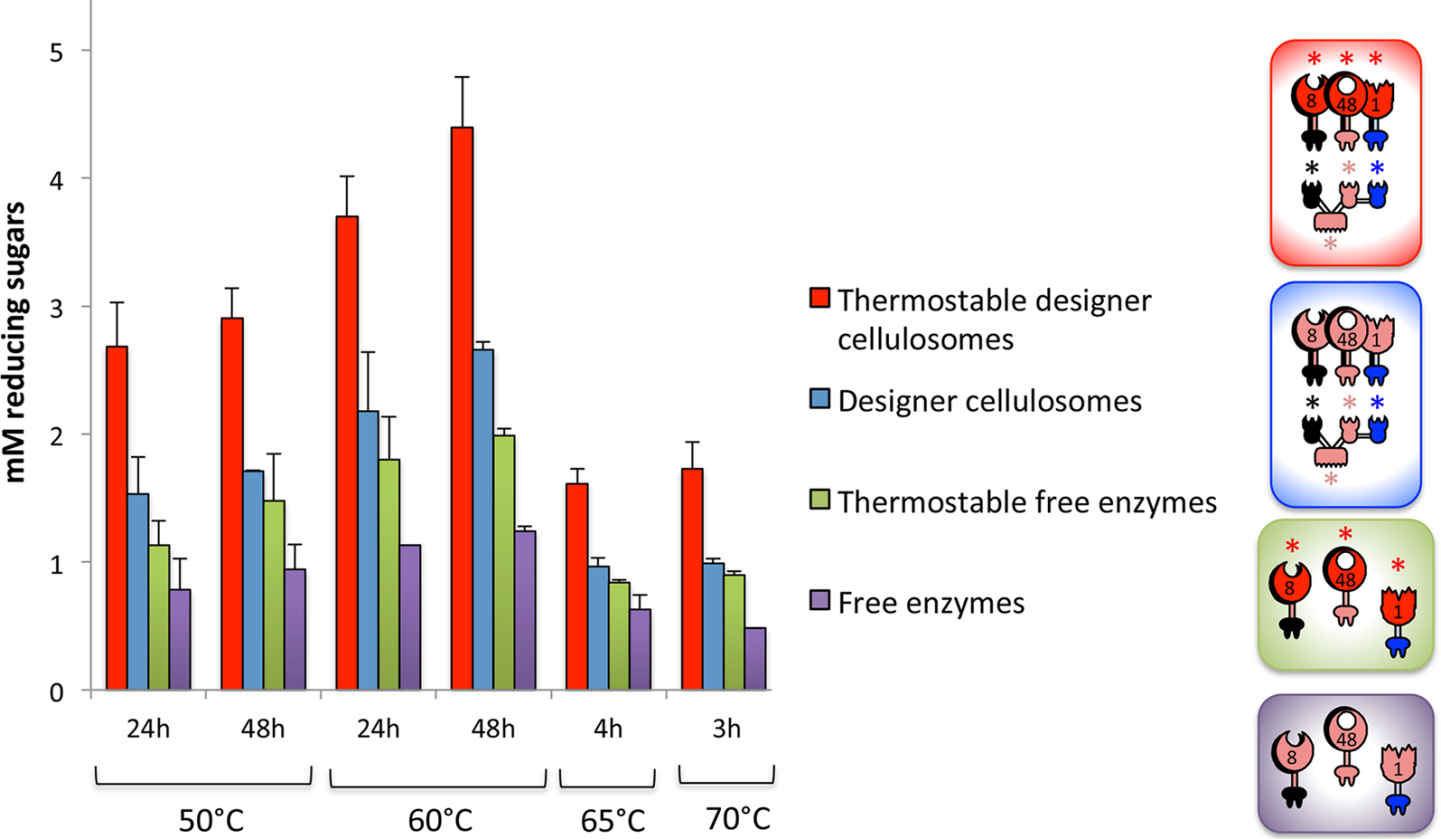
Comparative enzymatic activity of thermostable and conventional designer cellulosomes versus free enzymes. Thermostable designer cellulosomes consist of the thermostable scaffoldin that integrates the thermostable enzymes and conventional designer cellulosomes contain the wild-type enzymes. Experiments were performed on Avicel as a microcrystalline cellulose substrate, at the indicated time points and temperatures. Each reaction was performed in triplicate, and standard deviations are indicated. *Pictograms* denote composition of the designer cellulosomes and complementary free-enzyme cocktails. See Figs. 1 and 4 and legends for descriptions

**Fig. 6:**
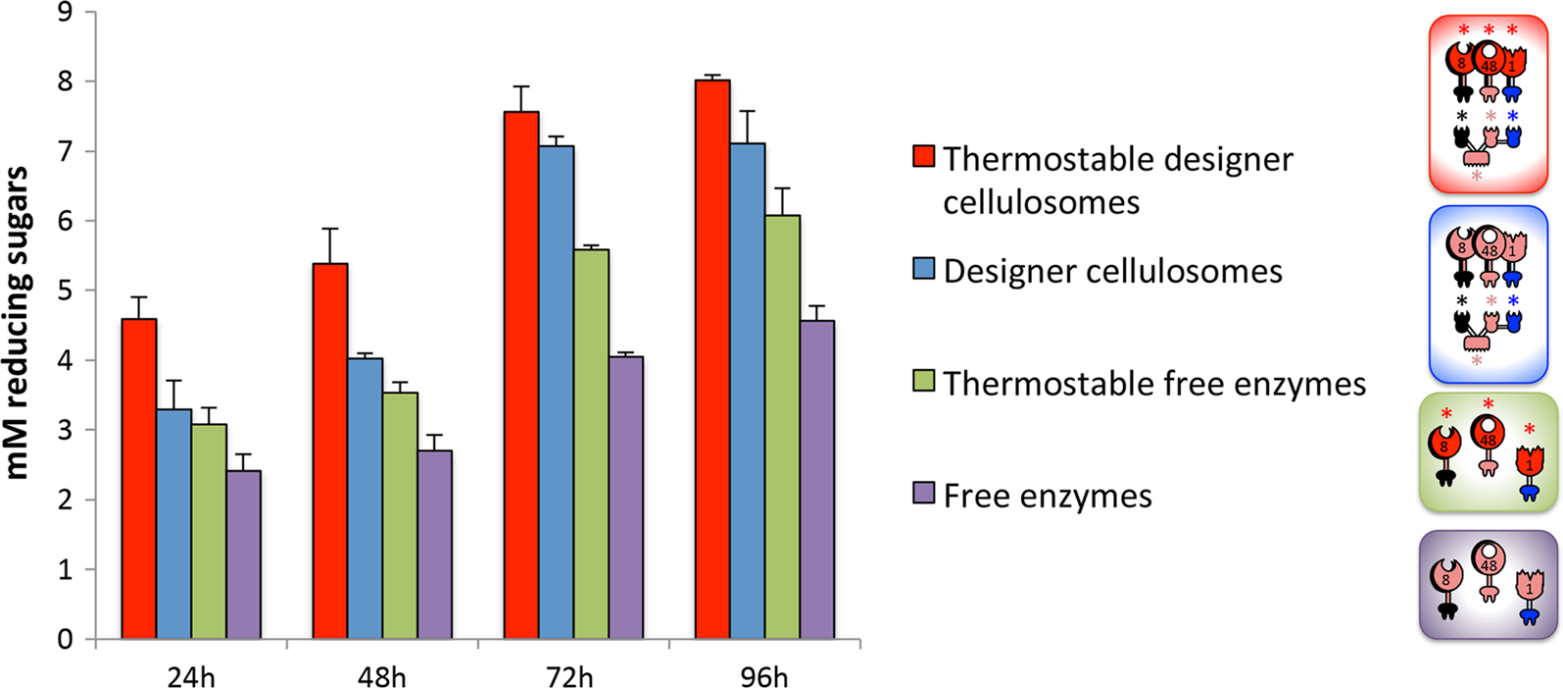
Comparative cellulolytic activity of designer cellulosomes versus free enzymes for extended time periods at 60 °C. Thermostable designer cellulosomes consist of the thermostable scaffoldin that integrates the thermostable enzymes and conventional designer cellulosomes contain the wild-type enzymes. Experiments were performed on Avicel from 24 to 96 h. Each reaction was performed in triplicate, and standard deviations are indicated.* Pictograms* denote composition of the designer cellulosomes and complementary free-enzyme cocktails. See Figs. 1 and 4 and legends for descriptions

Following stability studies of the different complexes, enzymatic activity of both thermostable and conventional (containing wild-type enzymes) designer cellulosomes was examined, and the results were compared to those of the mixtures of the free enzymes (without a scaffoldin) at various times and temperatures (corresponding to the predetermined maximum stability of the thermostable designer cellulosomes) on a microcrystalline cellulose substrate (Avicel). Both conventional designer cellulosomes and thermostable designer cellulosomes were consistently more active than the mixtures of their corresponding free enzymes. In addition, mixtures of the free thermostable enzymes continuously showed higher levels of cellulolysis than those of the wild-type free enzymes. Moreover, we observed that in each case the thermostable designer cellulosomes was advantageous (1.7 enhancement) in cellulose hydrolysis over that of conventional designer cellulosomes and free enzyme mixtures (Fig. 5). The main product of degradation was glucose, and the tendency in the measured concentrations after 24-h enzymatic degradation at 60 °C was similar to the total soluble concentrations as measured by the DNS assay (2.8 ± 0.4; 1.6 ± 0.2; 0.8 ± 0.05 and 0.6 ± 0.02 mM, for the thermostable designer cellulosomes, conventional designer cellulosomes, thermostable free enzymes and conventional free enzymes, respectively). These tendencies were conserved over the time range examined, upon incubating the samples for an additional 24 or 48 h (Fig. 6). Maximal hydrolysis was reached by conventional and thermostable designer cellulosomes after 72-h incubation, and a slight increase in hydrolysis was observed by the free enzymes from 72- to 96-h incubation.

## Discussion

Constant progress in understanding and improving enzymatic degradation of lignocellulose through the application of designer cellulosome complexes has established them as an attractive and promising tool for converting plant-derived cellulosic material into biofuels. To further reduce the costs of such an approach for industrial application, enzymes that maintain structural integrity above 50–55 °C would be desirable [16, 23]. In order to examine the relevance of this premise to designer cellulosomes, we selected three complementary thermostable cellulase mutants from *C. thermocellum* (endoglucanase Cel8A, exoglucanase Cel48S and β-glucosidase BglA). We compared the action of the combination of the native enzymes versus that of their thermostable mutants, either in the free state or in the context of designer cellulosome complexes. The selected cellulases exhibited enhanced synergistic effects in the cellulosome mode as compared to the free mode. In addition, thermostable designer cellulosomes had a clear advantage over conventional designer cellulosomes at various times and temperatures. The conventional complex contained the same thermostable scaffoldin subunit but the wild-type enzymes were incorporated therein instead of the thermostable mutated enzymes. Therefore, our results demonstrated that cellulosome-mediated deconstruction of cellulosic substrates can be improved by enhancing enzyme stability.

In a previous study [26], the enzymatic activity of the thermostable endoglucanase (8A*) was examined in the designer cellulosome mode in combination with the wild-type exoglucanase Cel48S. The resultant hybrid designer cellulosome between the thermostabilized enzyme and the wild-type enzyme failed to produce an increase in cellulose degradation. Consequently, in the present study, we tried to improve the performance of such artificial cellulosomes by including 3 thermostabilized enzymes together with a thermostable scaffoldin. It, therefore, seems that to enhance the efficiency of designer cellulosomes at elevated temperatures more than one component has to be engineered for increased thermostability. We used in the present study three complementary cellulases—the major *C. thermocellum* cellulosomal exoglucanase and endoglucanase plus the non-cellulosomal β-glucosidase—each converted to the cellulosomal mode. Each enzyme was mutated for increased thermostability, and the mutated enzymes were incorporated into a chimaeric scaffoldin, composed entirely of thermostable parts (all originating from thermophilic microbes). This scaffoldin proved, in fact, the most thermostable component of our thermostable designer cellulosomes. In addition, by using this extremely stable scaffoldin, thermostable designer cellulosomes could achieve enzymatic degradation for extended periods of time (96 h) at 60 °C and for short times at 65 and 70 °C. This was not the case when using a scaffoldin composed of a mixture of mesophilic and thermophilic modules (this study and [33]). In the latter case, the stability of the resultant designer cellulosomes was limited to 48 h at 50 °C and 6 h at 60 °C.

In future studies, the thermostable designer cellulosomes developed in the current work can be complemented with additional thermostable cellulases and hemicellulases. In addition to the cellulases engineered in this work for enhanced thermostability, thermostabilized cellulases from other families would presumably further improve degradation of cellulosic substrates. Moreover, xylanases from *C. thermocellum,* such as XynX or XynY that contain family 22 CBMs that were shown to confer thermostability to the catalytic modules [43, 44], could be further engineered for enhanced thermostability. Alternatively, wild-type hemicellulases from hyperthermophilic bacteria such as *Thermotoga maritima* [45] or thermophilic enzymes reported to exhibit enzymatic activities at extreme temperatures [46] could be converted to the cellulosomal mode and integrated into the thermostable designer cellulosomes.

In future extensions of this approach, it will be essential to increase our collection of thermostable cohesin and dockerin pairs. Thus far, we only know about the three thermophilic microbes that produce these basic cellulosomal components. We will have to either discover novel cellulosome-producing species, which may well prove challenging, or devise novel methodologies for improving the thermostability of existing cohesin–dockerin pairs. Finally, the thermostable designer cellulosomes described herein (the genes encoding for these enzymes together with that of the chimaeric scaffoldin) can be supplemented into solventogenic thermophiles for future construction of a combined consolidated-bioprocessing microorganism for direct biomass conversion to biofuels [13].

## Conclusions

Construction of efficient designer cellulosomes is a challenging prospect and has experienced continuous improvement throughout the past decade. The present report demonstrates that in addition to their multiple industrial advantages, thermostable cellulases and scaffoldins represent an important tool for application of designer cellulosomes for biomass degradation. However, mutagenic improvement of the properties of an individual enzyme does not necessarily ensure its performance as a component of a multienzyme composition. The true benefit of an engineered enzyme to synergistic substrate hydrolysis must be examined experimentally together with additional enzyme partners. Moreover, the thermostability properties of the scaffoldin component are critical to the integrity and performance of designer cellulosomes engineered for enhanced activity at elevated temperatures. Further research for the development of thermostable cellulosomes containing thermostable lignocellulosic enzymes and the stabilization at high temperatures of the chimaeric scaffoldins could lead to the development of more advanced designer cellulosomes, in view of their potential employment for cost-efficient conversion of cellulosic biomass to soluble sugars and biofuel production [22, 47].

## Additional files

10.1186/s13068-016-0577-z Non-denaturing PAGE analysis of wild-type and mutant forms of Cel48S complexed with the monovalent scaffoldin Scaf·T. Complexes were produced at different molar ratios and applied to non-denaturing polyacrylamide gels. For each gel, lanes 1 to 7 correspond to the respective enzyme/scaffoldin complexes at the following ratios: 0.4, 0.6, 0.8, 1.0, 1.2, 1.4 and 1.6. (Lane 4 signifies the calculated 1:1 ratio). Lane 8 corresponds to the specified enzyme alone at the presumed molar ratio. Red rectangle indicated the determined exact equimolar ratio.
10.1186/s13068-016-0577-z Comparative thermostability of wild-type β-glucosidase and BglA*. The enzymes were incubated at 66-72 °C for 1 h at pH 6.1. Residual activity was determined at a protein concentration of 1.05 µg/mL following incubation with 1 mM *p*NPG (Sigma-Aldrich, Israel) for 10 min at 60°C. Reactions were terminated by 1 M sodium carbonate and optical densities of the samples were measured at 405 nm (pNP released). Residual activities were calculated by dividing the activity of non-heated samples by that of the heated samples. The assay was performed at least twice in triplicate, error bars are indicated. Thermostable BglA* was generated by error-prone PCR as described by Anbar [24] using Gene-Morph II Random Mutagenesis Kit (Stratagene, La Jolla, CA). The thermostable BglA* was selected as it maintained the highest enzymatic activity after heat shock at 70 °C for 50 min (conditions in which the wild-type enzyme was no longer active). BglA* exhibited 127 % activity compared to the wild-type enzyme following heat shock at 66 °C for 75 min, and incubation with 1 mM *p*NPG for 45 min at 60 °C at 13 nM. Sequencing of the clone revealed 2 mutations: A17S and K268N.
10.1186/s13068-016-0577-z Thermostability of conventional versus thermostable designer cellulosomes and components. Non-denaturing PAGE (9 %) was employed for assessing total complex formation of the thermostable chimaeric scaffoldin and the wild-type or thermostable chimaeric enzymes into conventional designer cellulosomes (A) or thermostable designer cellulosomes (B), respectively, at the designated time periods and temperatures. In (C), the individual thermostable enzymes were incorporated into the thermostable scaffoldin. The stability of the trivalent scaffoldin either alone or in complex with each one of the thermostable dockerin-containing enzyme at various temperatures/times is shown. The tetravalent mesostable scaffoldin, bearing 3 thermostable cellulases was assayed similarly in (D). Pictograms denote composition of the designer cellulosomes and complementary free-enzyme cocktails. See Figures 1 and 4 and accompanying legends for descriptions of the pictograms.

## Abbreviations

Bgl: β-glucosidase
Cel: cellulase
CBM: carbohydrate binding module
DNS: dinitrosalicylic acid
Scaf: scaffoldin
GH: glycoside hydrolase
Xyn: xylanase

## References

[1] Himmel M, Xu Q, Luo Y, Ding S, Lamed R, Bayer EA (2010). Microbial enzyme systems for biomass conversion: emerging paradigms. Biofuels.

[2] Bayer EA, Belaich JP, Shoham Y, Lamed R (2004). The cellulosomes: multienzyme machines for degradation of plant cell wall polysaccharides. Annu Rev Microbiol.

[3] Lamed R, Setter E, Bayer EA (1983). Characterization of a cellulose-binding, cellulase-containing complex in *Clostridium thermocellum*. J Bacteriol.

[4] Bayer EA, Shimon LJ, Shoham Y, Lamed R (1998). Cellulosomes-structure and ultrastructure. J Struct Biol.

[5] Bayer EA, Morag E, Lamed R (1994). The cellulosome–a treasure-trove for biotechnology. Trends Biotechnol.

[6] Morais S, Morag E, Barak Y, Goldman D, Hadar Y, Lamed R, Shoham Y, Wilson DB, Bayer EA (2012). Deconstruction of lignocellulose into soluble sugars by native and designer cellulosomes. MBio.

[7] Stern J, Morais S, Lamed R, Bayer EA (2016). Adaptor scaffoldins: an original strategy for extended designer cellulosomes inspired from nature. MBio.

[8] Fierobe HP, Mingardon F, Mechaly A, Belaich A, Rincon MT, Pages S, Lamed R, Tardif C, Belaich JP, Bayer EA (2005). Action of designer cellulosomes on homogeneous versus complex substrates: controlled incorporation of three distinct enzymes into a defined trifunctional scaffoldin. J Biol Chem.

[9] Moraïs S, Barak Y, Caspi J, Hadar Y, Lamed R, Shoham Y, Wilson DB, Bayer EA (2010). Cellulase-xylanase synergy in designer cellulosomes for enhanced degradation of a complex cellulosic substrate. MBio..

[10] Caspi J, Barak Y, Haimovitz R, Irwin D, Lamed R, Wilson DB, Bayer EA (2009). Effect of linker length and dockerin position on conversion of a *Thermobifida fusca* endoglucanase to the cellulosomal mode. Appl Environ Microbiol.

[11] Mingardon F, Chanal A, Tardif C, Bayer EA, Fierobe H-P (2007). Exploration of new geometries in cellulosome-like chimeras. Appl Environ Microbiol.

[12] Arfi Y, Shamshoum M, Rogachev I, Peleg Y, Bayer EA (2014). Integration of bacterial lytic polysaccharide monooxygenases into designer cellulosomes promotes enhanced cellulose degradation. Proc Natl Acad Sci USA.

[13] Blumer-Schuette SE, Brown SD, Sander KB, Bayer EA, Kataeva I, Zurawski JV, Conway JM, Adams MW, Kelly RM (2014). Thermophilic lignocellulose deconstruction. FEMS Microbiol Rev.

[14] Shao W, Wiegel J (1995). Purification and characterization of two thermostable acetyl xylan esterases from *Thermoanaerobacterium sp*. strain JW/SL-YS485. Appl Environ Microbiol.

[15] Viikari L, Alapuranen M, Puranen T, Vehmaanpera J, Siika-Aho M (2007). Thermostable enzymes in lignocellulose hydrolysis. Adv Biochem Eng Biotechnol.

[16] Yeoman CJ, Han Y, Dodd D, Schroeder CM, Mackie RI, Cann IK (2010). Thermostable enzymes as biocatalysts in the biofuel industry. Adv Appl Microbiol.

[17] Yennamalli RM, Rader AJ, Kenny AJ, Wolt JD, Sen TZ (2013). Endoglucanases: insights into thermostability for biofuel applications. Biotechnol Biofuels.

[18] Anbar M, Bayer EA (2012). Approaches for improving thermostability characteristics in cellulases. Methods Enzymol.

[19] Szijarto N, Siika-Aho M, Tenkanen M, Alapuranen M, Vehmaanpera J, Reczey K, Viikari L (2008). Hydrolysis of amorphous and crystalline cellulose by heterologously produced cellulases of *Melanocarpus albomyces*. J Biotechnol.

[20] Stutzenberger F (1990). Thermostable fungal β-glucosidases. Lett Appl Microbiol.

[21] Haki GD, Rakshit SK (2003). Developments in industrially important thermostable enzymes: a review. Bioresour Technol.

[22] Arora R, Behera S, Sharma NK, Kumar S (2015). Bioprospecting thermostable cellulosomes for efficient biofuel production from lignocellulosic biomass. Bioresour Bioprocess.

[23] Bhalla A, Bansal N, Kumar S, Bischoff KM, Sani RK (2013). Improved lignocellulose conversion to biofuels with thermophilic bacteria and thermostable enzymes. Bioresour Technol.

[24] Anbar M, Gul O, Lamed R, Sezerman UO, Bayer EA (2012). Improved thermostability of *Clostridium thermocellum* endoglucanase Cel8A by using consensus-guided mutagenesis. Appl Environ Microbiol.

[25] Anbar M, Lamed R, Bayer E (2010). Thermostability enhancement of *Clostridium thermocellum* cellulosomal endoglucanase Cel8A by a single glycine substitution. ChemCatChem.

[26] Stern J, Anbar M, Morais S, Lamed R, Bayer EA (2014). Insights into enhanced thermostability of a cellulosomal enzyme. Carbohydr Res.

[27] Smith MA, Rentmeister A, Snow CD, Wu T, Farrow MF, Mingardon F, Arnold FH (2012). A diverse set of family 48 bacterial glycoside hydrolase cellulases created by structure-guided recombination. FEBS J.

[28] Zverlov VV, Kellermann J, Schwarz WH (2005). Functional subgenomics of *Clostridium thermocellum* cellulosomal genes: identification of the major catalytic components in the extracellular complex and detection of three new enzymes. Proteomics.

[29] Vazana Y, Barak Y, Unger T, Peleg Y, Shamshoum M, Ben-Yehezkel T, Mazor Y, Shapiro E, Lamed R, Bayer EA (2013). A synthetic biology approach for evaluating the functional contribution of designer cellulosome components to deconstruction of cellulosic substrates. Biotechnol Biofuels.

[30] Pallapolu VR, Lee YY, Garlock RJ, Balan V, Dale BE, Kim Y, Mosier NS, Ladisch MR, Falls M, Holtzapple MT (2011). Effects of enzyme loading and β-glucosidase supplementation on enzymatic hydrolysis of switchgrass processed by leading pretreatment technologies. Bioresour Technol.

[31] Hsieh CW, Cannella D, Jorgensen H, Felby C, Thygesen LG (2014). Cellulase inhibition by high concentrations of monosaccharides. J Agric Food Chem.

[32] Gefen G, Anbar M, Morag E, Lamed R, Bayer EA (2012). Enhanced cellulose degradation by targeted integration of a cohesin-fused β-glucosidase into the *Clostridium thermocellum* cellulosome. Proc Natl Acad Sci USA.

[33] Galanopoulou AP, Moraïs S, Georgoulis A, Morag E, Bayer EA, Hatzinikolaou DG. Insights into the functionality and stability of designer cellulosomes at elevated temperatures. Appl Microbiol Biotechnol. 2016:In press. 10.1007/s00253-016-7594-5 27207145

[34] Barak Y, Handelsman T, Nakar D, Mechaly A, Lamed R, Shoham Y, Bayer EA (2005). Matching fusion protein systems for affinity analysis of two interacting families of proteins: the cohesin-dockerin interaction. J Mol Recogn.

[35] Vazana Y, Morais S, Barak Y, Lamed R, Bayer EA (2010). Interplay between *Clostridium thermocellum* family 48 and family 9 cellulases in cellulosomal versus noncellulosomal states. Appl Environ Microbiol.

[36] Caspi J, Irwin D, Lamed R, Shoham Y, Fierobe H-P, Wilson DB, Bayer EA (2006). *Thermobifida fusca* family-6 cellulases as potential designer cellulosome components. Biocatal Biotransform.

[37] Morais S, David YB, Bensoussan L, Duncan SH, Koropatkin NM, Martens EC, Flint HJ, Bayer EA (2016). Enzymatic profiling of cellulosomal enzymes from the human gut bacterium, *Ruminococcus champanellensis*, reveals a fine-tuned system for cohesin-dockerin recognition. Environ Microbiol.

[38] Gasteiger E, Hoogland C, Gattiker A, Duvaud S, Wilkins MR, Appel RD, Bairoch A, Walker JM, Totowa NJ (2005). Protein identification and analysis tools on the ExPASy server. The proteomics protocols handbook.

[39] Miller GL (1959). Use of dinitrosalicylic acid reagent for determination of reducing sugar. AB.

[40] Morag E, Halevy I, Bayer EA, Lamed R (1991). Isolation and properties of a major cellobiohydrolase from the cellulosome of *Clostridium thermocellum*. J Bacteriol.

[41] Vazana Y, Moraïs S, Barak Y, Lamed R, Bayer EA (2012). Designer cellulosomes for enhanced hydrolysis of cellulosic substrates. ME.

[42] Morais S, Barak Y, Hadar Y, Wilson DB, Shoham Y, Lamed R, Bayer EA. Assembly of xylanases into designer cellulosomes promotes efficient hydrolysis of the xylan component of a natural recalcitrant cellulosic substrate. MBio. 2011;2. 10.1128/mBio.00233-11 PMC3221603 22086489

[43] Fontes CM, Hazlewood GP, Morag E, Hall J, Hirst BH, Gilbert HJ (1995). Evidence for a general role for non-catalytic thermostabilizing domains in xylanases from thermophilic bacteria. Biochem J.

[44] Shin ES, Yang MJ, Jung KH, Kwon EJ, Jung JS, Park SK, Kim J, Yun HD, Kim H (2002). Influence of the transposition of the thermostabilizing domain of *Clostridium thermocellum* xylanase (XynX) on xylan binding and thermostabilization. Appl Environ Microbiol.

[45] Jiang ZQ, Kobayashi A, Ahsan MM, Lite L, Kitaoka M, Hayashi K (2001). Characterization of a thermostable family 10 endo-xylanase (XynB) from *Thermotoga maritima* that cleaves p-nitrophenyl-β-D-xyloside. J Biosci Bioeng.

[46] Wu S, Liu B, Zhang X (2006). Characterization of a recombinant thermostable xylanase from deep-sea thermophilic *Geobacillus* sp. MT-1 in East Pacific. Appl Microbiol Biotechnol.

[47] Fontes CM, Gilbert HJ (2010). Cellulosomes: highly efficient nanomachines designed to deconstruct plant cell wall complex carbohydrates. Annu Rev Biochem.

[48] Artzi L, Dassa B, Borovok I, Shamshoum M, Lamed R, Bayer EA (2014). Cellulosomics of the cellulolytic thermophile *Clostridium clariflavum*. Biotechnol Biofuels.

[49] Morais S, Barak Y, Caspi J, Hadar Y, Lamed R, Shoham Y, Wilson DB, Bayer EA (2010). Contribution of a xylan-binding module to the degradation of a complex cellulosic substrate by designer cellulosomes. Appl Environ Microbiol.

