# Supplementary material for: Enhancement of cellulosome-mediated deconstruction of cellulose by improving enzyme thermostability
Source: Biotechnol Biofuels. 2016 Aug 4;9:164. doi: 10.1186/s13068-016-0577-z (PMC4973527; doi:10.1186/s13068-016-0577-z)
Supplement: Supplementary file 1 — 10.1186/s13068-016-0577-z Non-denaturing PAGE analysis of wild-type and mutant forms of Cel48S complexed with the monovalent scaffoldin Scaf·T. Complexes were produced at different molar ratios and applied to non-denaturing polyacrylamide gels. For each gel, lanes 1 to 7 correspond to the respective enzyme/scaffoldin complexes at the following ratios: 0.4, 0.6, 0.8, 1.0, 1.2, 1.4 and 1.6. (Lane 4 signifies the calculated 1:1 ratio). Lane 8 corresponds to the specified enzyme alone at the presumed molar ratio. Red rectangle indicated the determined exact equimolar ratio. [file 13068_2016_577_MOESM1_ESM.docx]

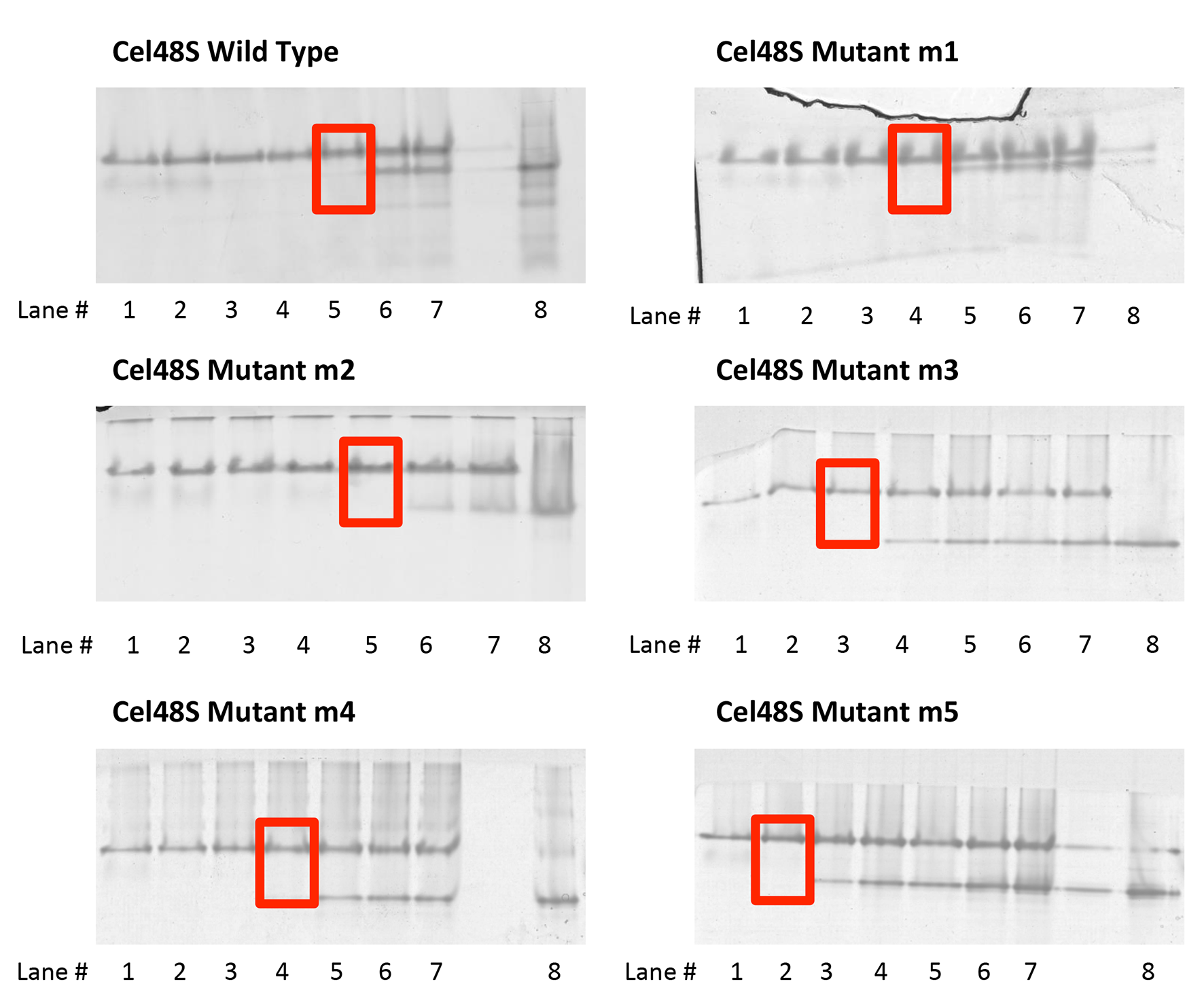


**Additional file 1.** Non-denaturing PAGE analysis of wild-type and mutant forms of Cel48S complexed with the monovalent scaffoldin Scaf·T. Complexes were produced at different molar ratios and applied to non-denaturing polyacrylamide gels. For each gel, lanes 1 to 7 correspond to the respective enzyme/scaffoldin complexes at the following ratios: 0.4, 0.6, 0.8, 1.0, 1.2, 1.4 and 1.6. (Lane 4 signifies the calculated 1:1 ratio). Lane 8 corresponds to the specified enzyme alone at the presumed molar ratio. Red rectangle indicated the determined exact equimolar ratio.
