# Supplementary material for: Enhancement of cellulosome-mediated deconstruction of cellulose by improving enzyme thermostability
Source: Biotechnol Biofuels. 2016 Aug 4;9:164. doi: 10.1186/s13068-016-0577-z (PMC4973527; doi:10.1186/s13068-016-0577-z)
Supplement: Supplementary file 2 — 10.1186/s13068-016-0577-z Comparative thermostability of wild-type β-glucosidase and BglA*. The enzymes were incubated at 66-72 °C for 1 h at pH 6.1. Residual activity was determined at a protein concentration of 1.05 µg/mL following incubation with 1 mM pNPG (Sigma-Aldrich, Israel) for 10 min at 60°C. Reactions were terminated by 1 M sodium carbonate and optical densities of the samples were measured at 405 nm (pNP released). Residual activities were calculated by dividing the activity of non-heated samples by that of the heated samples. The assay was performed at least twice in triplicate, error bars are indicated. Thermostable BglA* was generated by error-prone PCR as described by Anbar [24] using Gene-Morph II Random Mutagenesis Kit (Stratagene, La Jolla, CA). The thermostable BglA* was selected as it maintained the highest enzymatic activity after heat shock at 70 °C for 50 min (conditions in which the wild-type enzyme was no longer active). BglA* exhibited 127 % activity compared to the wild-type enzyme following heat shock at 66 °C for 75 min, and incubation with 1 mM pNPG for 45 min at 60 °C at 13 nM. Sequencing of the clone revealed 2 mutations: A17S and K268N. [file 13068_2016_577_MOESM2_ESM.docx]

**
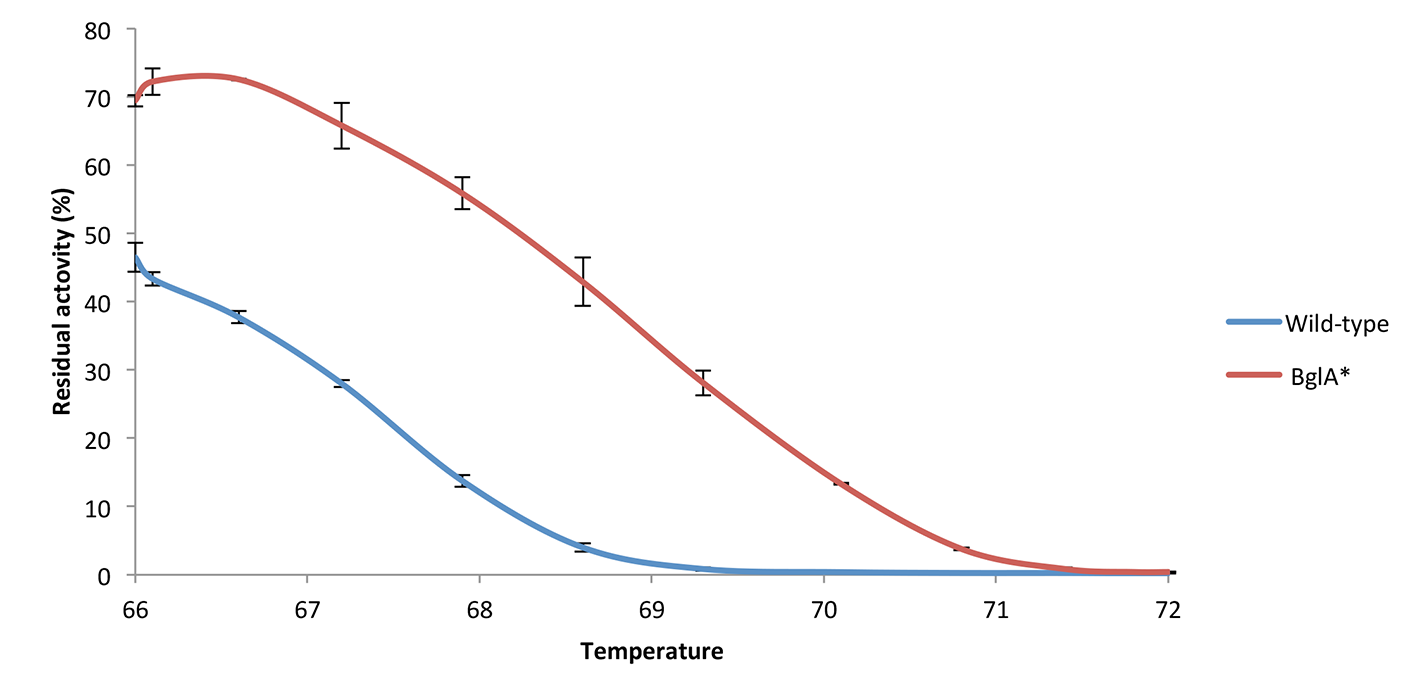
**

**Additional file 2.** Comparative thermostability of wild-type β-glucosidase and BglA*. The enzymes were incubated at 66-72°C for 1 h at pH 6.1. Residual activity was determined at a protein concentration of 1.05 µg/mL following incubation with 1 mM *p*NPG (Sigma-Aldrich, Israel) for 10 min at 60°C. Reactions were terminated by 1 M sodium carbonate and optical densities of the samples were measured at 405 nm (pNP released). Residual activities were calculated by dividing the activity of non-heated samples by that of the heated samples. The assay was performed at least twice in triplicate, error bars are indicated.

Thermostable BglA* was generated by error-prone PCR as described by Anbar [[24](#_ENREF_24)] using Gene-Morph II Random Mutagenesis Kit (Stratagene, La Jolla, CA). The thermostable BglA* was selected as it maintained the highest enzymatic activity after heat-shock at 70°C for 50 min (conditions in which the wild-type enzyme was no longer active). BglA* exhibited 127% activity compared to the wild-type enzyme following heat shock at 66°C for 75 min, and incubation with 1 mM *p*NPG for 45 min at 60°C at 13 nM. Sequencing of the clone revealed 2 mutations: A17S and K268N.
