# Supplementary material for: Enhancement of cellulosome-mediated deconstruction of cellulose by improving enzyme thermostability
Source: Biotechnol Biofuels. 2016 Aug 4;9:164. doi: 10.1186/s13068-016-0577-z (PMC4973527; doi:10.1186/s13068-016-0577-z)
Supplement: Supplementary file 3 — 10.1186/s13068-016-0577-z Thermostability of conventional versus thermostable designer cellulosomes and components. Non-denaturing PAGE (9 %) was employed for assessing total complex formation of the thermostable chimaeric scaffoldin and the wild-type or thermostable chimaeric enzymes into conventional designer cellulosomes (A) or thermostable designer cellulosomes (B), respectively, at the designated time periods and temperatures. In (C), the individual thermostable enzymes were incorporated into the thermostable scaffoldin. The stability of the trivalent scaffoldin either alone or in complex with each one of the thermostable dockerin-containing enzyme at various temperatures/times is shown. The tetravalent mesostable scaffoldin, bearing 3 thermostable cellulases was assayed similarly in (D). Pictograms denote composition of the designer cellulosomes and complementary free-enzyme cocktails. See Figures 1 and 4 and accompanying legends for descriptions of the pictograms. [file 13068_2016_577_MOESM3_ESM.docx]

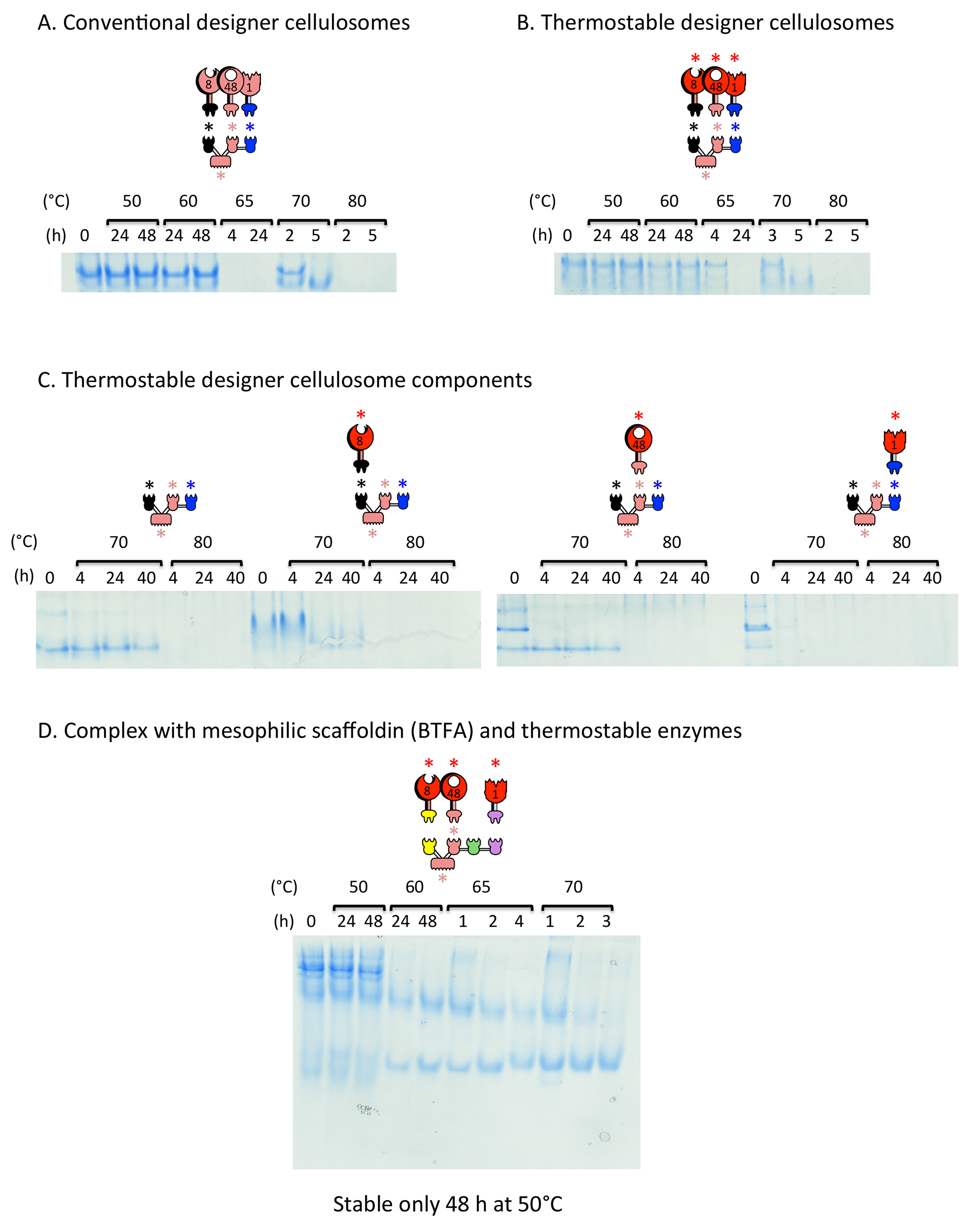


**Additional file 3.** Thermostability of conventional versus thermostable designer cellulosomes and components. Non-denaturing PAGE (9%) was employed for assessing total complex formation of the thermostable chimaeric scaffoldin and the wild-type or thermostable chimaeric enzymes into conventional designer cellulosomes (A) or thermostable designer cellulosomes (B), respectively, at the designated time periods and temperatures. In (C), the individual thermostable enzymes were incorporated into the thermostable scaffoldin. The stability of the trivalent scaffoldin either alone or in complex with each one of the thermostable dockerin-containing enzyme at various temperatures/times is shown. The tetravalent mesostable scaffoldin, bearing 3 thermostable cellulases was assayed similarly in (D). Pictograms denote composition of the designer cellulosomes and complementary free-enzyme cocktails. See Figures 1 and 4 and accompanying legends for descriptions of the pictograms.
